# Structural basis of L-glucose oxidation by *scyllo*-inositol dehydrogenase: Implications for a novel enzyme subfamily classification

**DOI:** 10.1371/journal.pone.0198010

**Published:** 2018-05-25

**Authors:** Kazuhiro Fukano, Kunio Ozawa, Masaya Kokubu, Tetsu Shimizu, Shinsaku Ito, Yasuyuki Sasaki, Akira Nakamura, Shunsuke Yajima

**Affiliations:** 1 Department of Bioscience, Tokyo University of Agriculture, Setagaya-ku, Tokyo, Japan; 2 Faculty of Life and Environmental Sciences, University of Tsukuba, Ibaraki, Japan; Universidade Nova de Lisboa Instituto de Tecnologia Quimica e Biologica, PORTUGAL

## Abstract

For about 70 years, L-glucose had been considered non-metabolizable by either mammalian or bacterial cells. Recently, however, an L-glucose catabolic pathway has been discovered in *Paracoccus laeviglucosivorans*, and the genes responsible cloned. *Scyllo*-inositol dehydrogenase is involved in the first step in the pathway that oxidizes L-glucose to produce L-glucono-1,5-lactone with concomitant reduction of NAD^+^ dependent manner. Here, we report the crystal structure of the ternary complex of *scyllo*-inositol dehydrogenase with NAD^+^ and L-glucono-1,5-lactone at 1.8 Å resolution. The enzyme adopts a homo-tetrameric structure, similar to those of the inositol dehydrogenase family, and the electron densities of the bound sugar was clearly observed, allowing identification of the residues responsible for interaction with the substrate in the catalytic site. In addition to the conserved catalytic residues (Lys106, Asp191, and His195), another residue, His318, located in the loop region of the adjacent subunit, is involved in substrate recognition. Site-directed mutagenesis confirmed the role of these residues in catalytic activity. We also report the complex structures of the enzyme with *myo*-inositol and *scyllo*-inosose. The Arg178 residue located in the flexible loop at the entrance of the catalytic site is also involved in substrate recognition, and plays an important role in accepting both L-glucose and inositols as substrates. On the basis of these structural features, which have not been identified in the known inositol dehydrogenases, and a phylogenetic analysis of IDH family enzymes, we suggest a novel subfamily of the GFO/IDH/MocA family. Since many enzymes in this family have not biochemically characterized, our results could promote to find their activities with various substrates.

## Introduction

Homochirality, in the form of L-amino acids and D-sugars, exists in all living organisms In the case of sugars, it had been a long held belief, first reported in 1940 by Rudney, that L-glucose cannot be metabolized by either mammalian or bacterial cells [1]. Subsequently, Sasajima et al. purified D-threo-aldose dehydrogenase from *Pseudomonas caryophylli*, which was capable of oxidizing L-glucose [2]. Recently, a catabolic pathway that can utilize L-glucose has been discovered in *Paracoccus laeviglucosivorans* [3], and the component genes have been cloned and characterized [4]. This pathway is made up by the combination of genes originating from two independent operons. *lgdA*, which codes for a protein that works at the first step, is located in a putative inositol catabolic gene cluster. The genes that code for proteins that work at the later steps in the pathway, *lgnE*, *lgnF*, *lgnG*, *lgnH*, and *lgnI* are located in an operon, which is analogous to *E*. *coli* L-galactonate catabolic pathway [5]. Using the combined pathway, L-glucose is metabolized to pyruvate and glyceraldehyde-3-phosphate.

The *lgdA* gene encodes inositol dehydrogenase, and on the basis of its amino acid sequence, the enzyme belongs to the glucose-fructose oxidase/inositol dehydrogenase/microbial rhizopine-catabolizing (GFO/IDH/MocA) family (Pfam ID: PF01408). Approximately 50 crystal structures of GFO/IDH/MocA family members, defined by their Pfam entry, have been deposited in PDB, most of them being reported with only coordinates as a result of structural genomics projects. The reported structures show homo-dimeric or homo-tetrameric conformations, and each subunit consists of two domains, an NAD^+^-binding domain at the N-terminus and a substrate-binding domain at the C-terminus. Several crystal structures have also been published, including GFOR from *Zymmomonas mobilis* [6, 7], *myo*-inositol dehydrogenase (*myo*-IDH) from *Bacillus subtilis* [8], and 1,5-anhydro-D-fructose reductase from *Sinorhizobium* [9]. As an enzyme using inositols as substrates, *myo*-IDH from *B*. *subtilis* has been studied both kinetically and by mutational analysis. Structures complexed with *myo*-inositol and inosose at 2.5 Å and 2.9 Å resolution, respectively, have also been reported [8].

**Fig 1:**
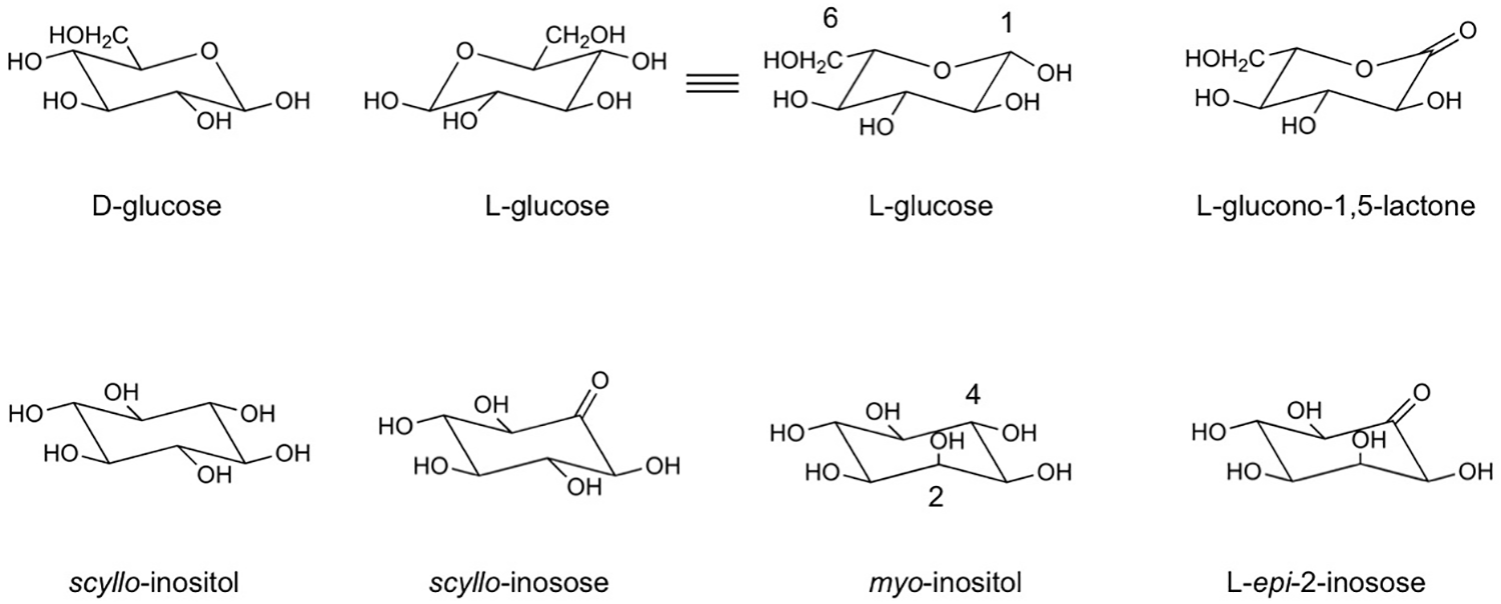
Structures of enzyme substrates and products. Numbers shown at *myo*-inositol and L-glucose denote the positions of hydroxyl group.

The protein encoded by *lgdA* from *P*. *laeviglucosivorans* was initially purified based on its ability to catalyze L-glucose dehydrogenation. Subsequently, it was considered a *scyllo*-inositol dehydrogenase with wide substrate spectrum catalyzing oxidation of *scyllo*-inositol, *myo*-inositol, and weakly D-glucose (Fig 1), among which *scyllo*-inositol seems to be the preferred substrate. The enzyme, therefore, turned out to be *scyllo*-inositol dehydrogenase with L-glucose dehydrogenase activity (*P*. *laeviglucosivorans scyllo*-inositol dehydrogenase; Pl-*scyllo*-IDH). Further analysis showed that the enzyme oxidized L-glucose to L-glucono-1,5-lactone (Fig 1) as the first step in the pathway for L-glucose metabolism [4]. Since L-glucose is not found in the natural environment, the principle role of Pl-*scyllo*-IDH in nature is very unlikely to be L-glucose oxidation. To elucidate how Pl-*scyllo*-IDH is able to recognize and oxidize L-glucose, we solved the crystal structure of Pl-*scyllo*-IDH complexed with several sugars, including L-glucose, *scyllo*-inosose, and *myo*-inositol. In addition, we performed site directed mutagenesis of Pl-*scyllo*-IDH, based on the crystal structures determined here, to confirm the catalytic mechanism. While there are many structures deposited in PDB that are annotated as being inositol dehydrogenases, few, however, have demonstrated enzymatic activity. Therefore, our structural analysis and mutagenesis study could provide important insights into the structure-activity relationship in the GFO/IDH/MocA family, and contribute to handling various kinds of sugars.

## Materials and methods

### Protein expression and purification

The *lgdA* gene was cloned into pET21a(+) and the recombinant protein was expressed in *Escherichia coli* BL21(DE3), as previously described [4]. *E*. *coli* harboring the plasmid was cultured in LB medium containing 50 μg/mL of ampicillin at 25°C. The protein production was induced by the addition of isopropyl-thio-β-galactopyranoside and the culture was continued for 14 hr at 25°C. The cells were pelleted and disrupted by sonification, and the supernatant was loaded onto a Ni-NTA agarose column. The column was washed with buffer A [20 mM Tris-HCl, pH 7.5, 10% glycerol] containing 20 mM imidazole, and the recombinant protein was eluted with buffer A containing 500 mM imidazole. The eluted protein was then dialyzed against buffer A. The collected fraction was concentrated to 10 mg/mL with an Amicon Ultra 10K prior to crystallization. To obtain the selenomethionine-substituted protein, *E*. *coli* B834(DE3) was used as the host strain with the LeMaster medium [10, 11] supplemented with thiamine (40 mg/L), and buffer A containing 1 mM and 10 mM DTT was used for the Ni-column purification and protein concentration, respectively.

### Crystallization and structure determination

Crystals were grown at 20°C by the hanging-drop vapor-diffusion method, with a drop consisting of 2.0 μL of protein solution and 2.0 μL of a crystallization reservoir solution containing 0.1 M sodium acetate pH 4.8–5.4, and 12–35% polyethylene glycol (PEG) 3350. Under these conditions, both native and Se-Met crystals were obtained, and these belonged to the space group *P*2_1_2_1_2_1_. Prior to data collection, the crystal was transferred for 1 min into a cryoprotectant solution [35%(w/v) PEG3350 in the reservoir solution], and flash-cooled in a liquid-nitrogen gas stream at 95 K. Diffraction data were collected on beamline 5A equipped with a Quantum 315 CCD detector and beamline AR BL-NW12A equipped with a Quantum 210 CCD detector (Area Detector Systems Corp., CA) at the Photon Factory, High Energy Accelerator Research Organization, Tsukuba, Japan. The diffraction data were processed using the HKL2000 package [12].

The initial phase was determined by the Se-SAD method and the structure of Se-Met Pl-*scyllo*-IDH was solved using AutoSol in Phenix [13]. The native apo-structure was then solved by the molecular replacement method, using the MORLEP software [14] in the CCP4 suite [15] with the Se-Met-structure as the search model. The native apo-structure was then used as a search model for other Pl-*scyllo*-IDH structures in complexes with substrates. Iterative model refinements were carried out using Coot [16] and REFMAC5 [17]. The coordinates of L-glucono-1,5-lactone were prepared using Discovery Studio (BIOVIA), and the topology file was produced by the PRODRG server [18]. The data collection and refinement statistics are presented in Table 1.

**Table 1:** Data collection and refinement statistics.

	Se-Met	Apo (acetate)	lactone/NADH	*myo*-inositol/NAD^+^	*scyllo*-inosose/NAD^+^
**Data collection**					
Beamline	PF BL-5A	PF BL-5A	PF BL-5A	PF-AR BL-NW12A	PF-AR BL-NW12A
Wavelength (Å)	0.97902	1.0000	1.0000	1.0000	1.0000
Detector	ADSC Q210			ADSC Q210	
Space group	P212121				
Unit cell parameters (Å)	90.5, 126.9, 137.9	91.9, 127.5, 137.5	86.6, 121.2, 140.9	86.2, 120.1, 140.6	86.6, 121.2, 140.4
Resolution range (Å)	27.3–1.9 (1.93–1.9)	28.3–1.75 (1.81–1.75)	29.5–1.8 (1.83–1.8)	49.3–2.3 (2.34–2.3)	39.0–2.0 (2.03–2.0)
No. of unique reflections	124012 (6130)	160030 (15041)	137356 (6735)	64176 (3102)	100761 (4956)
Redundancy	3.7 (3.7)	3.7 (3.1)	3.6 (3.4)	3.6 (3.6)	4.9 (4.7)
*R*_merge_	0.111 (0.564)	0.055 (0.247)	0.082 (0.619)	0.203 (0.952)	0.074 (0.381)
*I*/σ*I*	17.0 (2.6)	24.7 (4.9)	18.1 (2.3)	7.8 (1.8)	20.9 (3.4)
Completeness (%)	99.8 (99.9)	99.3 (94.4)	99.6 (99.0)	98.3 (95.8)	99.8 (99.9)
**Refinement**					
Resolution range (Å)					
No. of reflections		151942	130434	60883	95631
Completeness (%)		99.3 (93.2)	99.4 (96.6)	98.0 (92.8)	99.5 (95.9)
*R*_work_/*R*_free_		0.177/0.192 (0.218/0.218)	0.163/0.170 (0.232/0.249)	0.192/0.224 (0.259/0.303)	0.176/0.193 (0.217/0.221)
No. of non-H atoms					
Protein		11195	11125	11117	11117
Water		852	856	423	470
Substrate/cofactor		16 (Acetate)	36/176	36/176	36/176
R.m.s. deviation					
Bonds (Å)		0.009	0.010	0.014	0.013
Angle (°)		1.356	1.486	1.655	1.662
Average B factors (Å^2^)					
Protein		14.8	19.4	2.0	23.2
Water		20.1	26.8	30.9	26.6
Substrate/cofactor		14.3 (Acetate)	21.3/20.7	24.0/28.8	22.7/34.0
Ramachandran plot					
Favored (%)		98.4	98.4	96.9	97.5
Allowed (%)		1.6	1.6	2.8	2.3
Disallowed (%)		0.0	0.1	0.3	0.3

For determination of Pl-*scyllo*-IDH structures in complexes with substrates, crystals were soaked for one minute with 25 mM of the oxidized form of nicotinamide adenine dinucleotide, (NAD^+^) (Sigma-Aldrich Co., MO) and 100 mM substrate in the reservoir solution. L-Glucose and *myo*-inositol were purchased from Sigma-Aldrich, and *scyllo*-inosose was from the Hokko Chemical Industry Co., Ltd. (Tokyo, Japan), respectively. Figures for all protein structures were generated using PyMOL (Schrödinger, Inc., MA). The atomic coordinates and structure factors for the apo, L-glucono-1,5-lactone/NADH, *myo*-inositol/NAD^+^ and *scyllo*-inosose/NAD^+^ complexes have been deposited in the Protein Data Bank under the accession codes 5YAB, 5YAP, 5YA8 and 5YAQ, respectively.

### Mutant preparation

Mutants of Pl-*scyllo*-IDH containing single amino acid substitutions with alanine (K106A, D191A, H195A, R178A, and H318A) were prepared using a PrimeSTAR mutagenesis kit (Takara Bio, Shiga, Japan) according to the manufacturer’s protocol, with pET21a(+)-*lgdA* as the template. The primers used for construction of each mutant genes are shown in Table 2. Mutant enzymes were expressed and purified as described for the wild-type enzyme.

**Table 2:** Primers used for mutation.

K106A-f	CTGGAAGCGCCCATGGCGCTGAGCGTC
K106A-r	CATGGGCGCTTCCAGCCAGACATGCTT
D191A-f	CTGGGGGCTCTGGGCTGCCATCTGGTC
D191A-r	GCCCAGAGCCCCCAGCGCGCCCAGTCC
H195A-f	GGCTGCGCGCTGGTCAGCGTGATGGTG
H195A-r	GACCAGCGCGCAGCCCAGATCCCCCAG
R178A-f	AGCTGGGCGCTGACCCGCAAGGATGGT
R178A-r	GGTCAGCGCCCAGCTCCATGGCAGGTC
H318A-f	GGCGGCGCTAATTTCGGCTTCAACGAA
H318A-r	GAAATTAGCGCCGCCACCGGGGCAGAA

The lower case letters, f and r, in primers names denote forward and reverse, respectively. Nucleotide sequences are presented as the 5’ to 3’ direction.

### Enzyme assay of Pl-*scyllo*-IDH

Purified proteins were assayed for enzyme activity. The reaction mixture comprised of 100 mM Tris-HCl, pH 9.0, 20 mM NAD^+^, and a substrate in a total volume of 900 μL. The concentrations of substrate were 12.5, 25, 50, and 100 mM. Fifty microliter of 0.2 mg mL^-1^ enzyme solution was added, and the activities were measured by monitoring absorption at 340 nm using a V-750 spectrophotometer (Jasco corp., Tokyo, Japan). Kinetic parameters were calculated by the Michaelis-Menten equation using the software supplied with the spectrophotometer.

Kinetic parameter of Pl-*scyllo*-IDH for L-*epi*-2-inosose (Hokko Chemical Industry, Co., Ltd.) was measured in 190 μL reaction mixture comprised 100 mM MES-KOH, pH6.0, 200 mM NADH, and various concentration of L-*epi*-2-inosose: 1.25, 2.5, 5, 10, 20, and 40 mM at 25°C. Reactions were started by addition of 10 μL of 42 μg mL^-1^ enzyme solution, and the activities were measured by monitoring the decrease in absorption at 340 nm by using DU800 spectrophotometer (Beckman Coulter, Inc., CA). Data obtained were fit to the Michaelis-Menten equation using the non-linear regression tool of Origin 6.0 (Light Stone, Tokyo, Japan).

### Phylogenetic analysis

The data set comprised of 44 sequences from the PDB, annotated by Pfam as being GFO/IDH/MocA family members. The tree was constructed by the neighbor-joining method with the MEGA7 programs [19]. Bootstrap values were calculated from 1000 repeats.

## Results and discussion

### Overall structure of LGDH

We have solved four different Pl-*scyllo*-IDH structures, which are the apo-form, a complex with L-glucono-1,5-lactone (described below), a complex with *myo*-inositol, and a complex with *scyllo*-inosose. All the crystals belonged to the space group *P*2_1_2_1_2_1_ with unit cell parameters of *a* = 91.0, *b* = 127.5, *c* = 137.5 Å for the apo-structure and *a* = 86.6, *b* = 121.2, *c* = 140.9 Å for the complex structures. The apo-Pl-*scyllo*-IDH structure was refined at 1.75 Å, and the other complex structures ranged from 1.8–2.3 Å, depending on the substrate.

**Fig 2:**
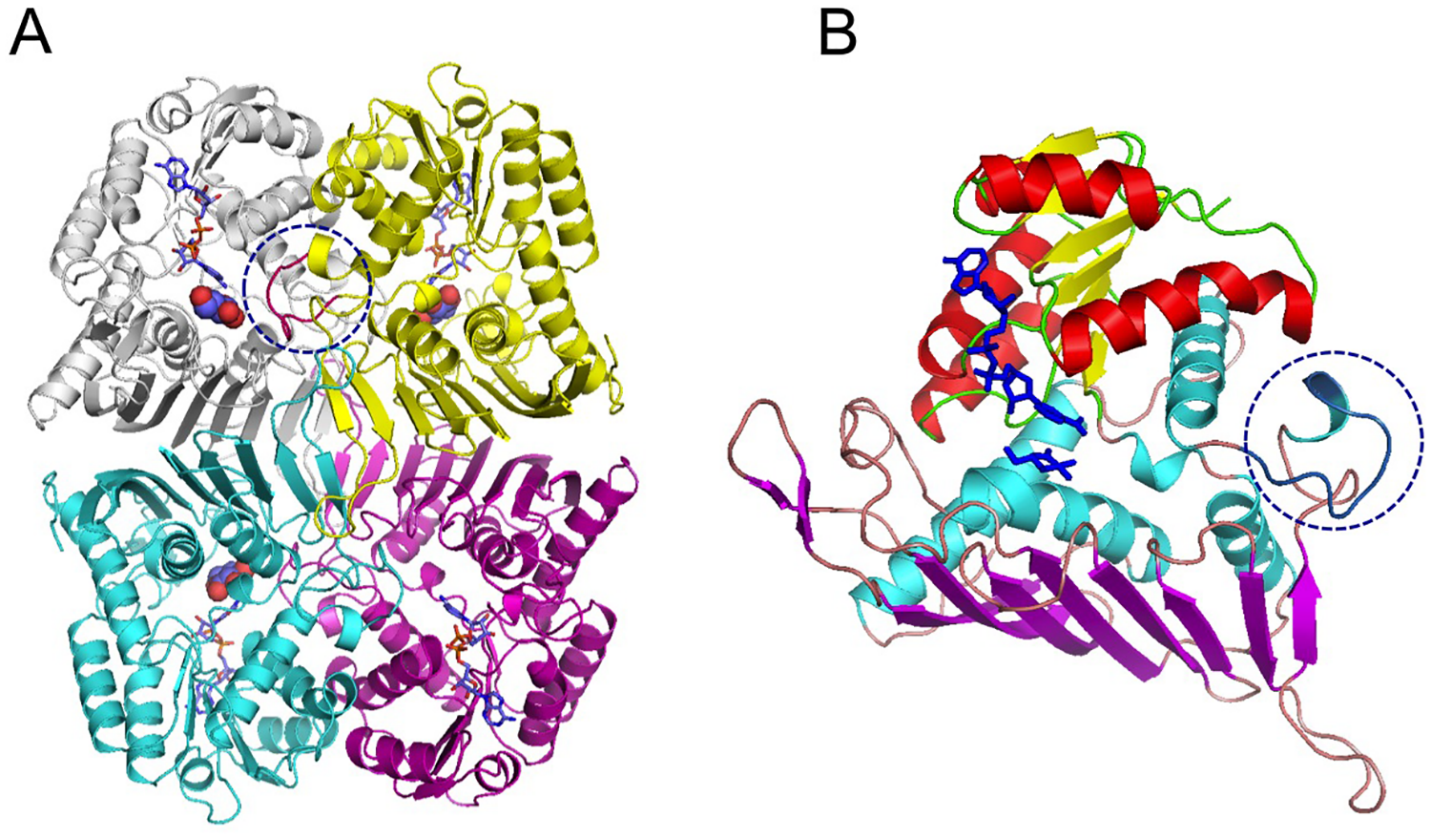
Overall structure of Pl-*scyllo*-IDH. (A) The overall structure of Pl-*scyllo*-IDH is shown as ribbon model. The structure adopts a homotetrameric conformation. In the structure, L-glucono-1,5-lactone and NADH as shown as sphere and stick models, respectively. A loop region colored in red in the yellow colored subunit protrudes to the active site in the next grey colored subunit, circled with a blue dotted line, described in detail in the text. (B) Subunit structure of Pl-*scyllo*-IDH shown as a ribbon model. Colors are assigned based on the secondary structure. NADH-binding and substrate-binding domains are colored yellow with red, and magenta with cyan, respectively. L-glucono-1,5-lactone and NADH are shown as stick models colored in blue. The loop region extending to the next subunit is marked with a blue dotted circle, which also corresponds to the circle in panel A.

Pl-*scyllo*-IDH formed a homo-tetrameric structure in the asymmetric unit (Fig 2A), and this structure was considered the biologically relevant form, since a similar unit has been observed for *myo*-IDH from *B*. *subtilis* [8] and GFOR from *Zymmomonas mobilis* [6].

As was observed for the other GFO/IDH/MocA structures, one Pl-*scyllo*-IDH subunit contained two domains (Fig 2B). The N-terminal domain adopted a Rossmann fold motif for the binding of NAD^+^, and the C-terminal domain bound substrate. The C-terminal domain contained a large β-sheet composed of 8 parallel and anti-parallel β-strands.

A long loop extending from G303 –G321 was found here to be a characteristic feature of Pl-*scyllo*-IDH (Fig 2A), which has not been found in other known *myo*-IDHs. This loop is involved in subunit interactions, as well as substrate recognition in the adjacent subunit, as described below. Other interactions involved in oligomer formation were mainly mediated by the 8-stranded β-sheet in the C-terminal domain.

A 3D structure comparison using the Dali server [20] revealed that the top hit in PDB was 4FB5 (Z = 52.0, rmsd = 1.1 Å) followed by 4H3V, 4GQA, 3V5N, 3DTY, and 2GLX, as well as others. The top five structures are biochemically uncharacterized, but commonly have loops extending to the active site in the adjacent subunit, as was seen here in Pl-*scyllo*-IDH. On the other hand, the hit structures listed below of 3DTY do not possess such a loop structure. 2GLX is 1,5-anhydro-D-fructose reductase (1,5-AFR) from *Sinorhizobium morelense* [9], followed by other IDHs mainly annotated by amino acid sequence homology. GFOR from *Zymmomonas mobilis* [6] was listed at the 13^th^ place in the non-redundant list with a Z-score and rmsd of 35.6 and 2 Å, respectively. *myo*-IDH from *B*. *subtilis*, whose activity has been biochemically confirmed [21], was listed at the 43^rd^ place with a Z-score and rmsd of 30.4 and 2.7 Å, respectively.

### L-glucose recognition by Pl-*scyllo*-IDH

To elucidate the L-glucose recognition mechanism, the crystal was soaked with L-glucose and the resulting structure was determined. The structure was refined at 1.8 Å, and the electron density of the bound sugar molecule was clearly observed. Among the four subunits, apparent electron density due to the substrate was observed in only three of the subunits. This observation was also seen in the two complex structures containing inositols, as described in the next section. The molecule in the active site fit in the density with a correct orientation, since L-glucose has a C6-hydroxyl group, making it easy to locate the positions of each hydroxyl group. As a result, the C1-hydroxyl group was seen to come into close contact (2.9 Å distance) to the Nζ atom of K106, which is likely to be one of the catalytic residues. This is in accordance with the result obtained by NMR analysis, which revealed L-gluconate, which was considered to be a compound spontaneously hydrolyzed from L-glucono-1,5-lactone [4].

**Fig 3:**
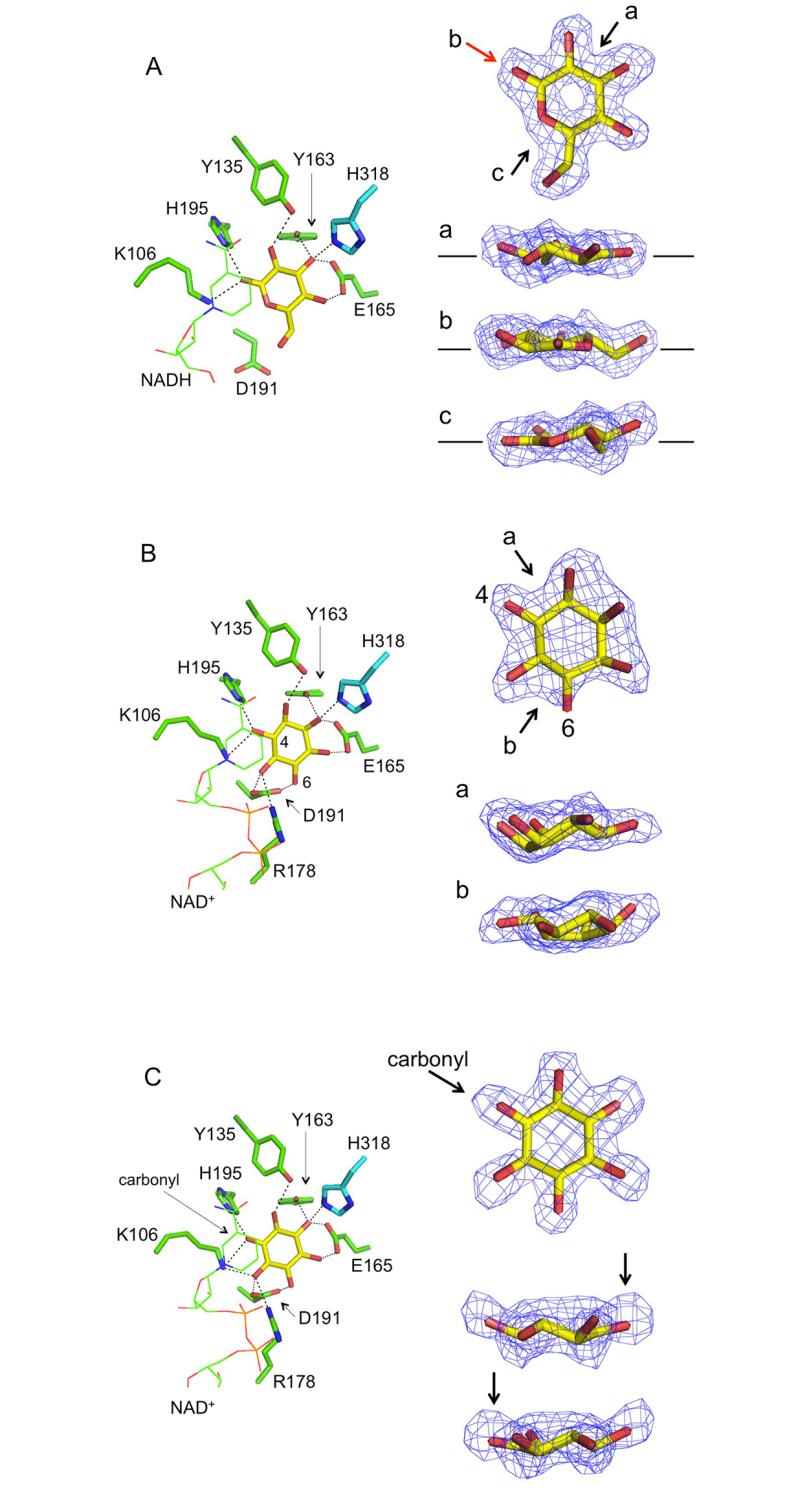
Binding modes of Pl-*scyllo*-IDH substrates. The binding modes of L-glucono-1,5-lactone (A), *myo*-inositol (B) and *scyllo*-inosose (C) are shown as stick models. The substrates and amino acids are colored yellow and green, respectively. His318, colored in cyan, belongs to the adjacent subunit. NAD^+^/NADH are also shown in a wireframe model colored in green. The dotted lines indicate the distances below 3.2 Å, with the exception of the 3.4 Å distance between Y163 and O2 in *myo*-inositol (B). Electron density of the Fo-Fc omit map contoured at 2σ, calculated excluding corresponding molecules, are also shown at the right side in each panel. In (A) and (B), right panels, the arrows indicate the viewing directions of the models, and the labels corresponds to the top in each panel. The red colored arrow b in (A) indicates a carbonyl group. In (C), the arrows show the position of a carbonyl group in *scyllo*-inosose.

Furthermore, based on the Fo-Fc omit map of the bound molecule, its C1, O1, C2 and O5 atoms were located on an identical plane, and the C1-hydroxyl group adopted neither an equatorial nor an axial conformation. We therefore concluded that the bound molecule in the active site was in fact the reaction product of L-glucose oxidation, namely L-glucono-1,5-lactone, despite the fact that the crystal had been soaked with L-glucose for data collection (Fig 3A). Accordingly, although we added NAD^+^ for crystallization, the bound cofactor molecule might therefore be NADH. The plane of the nicotinamide ring was parallel to the plane of the lactone ring, and C4 atom of the nicotinamide ring was located just above the C1 atom of the lactone at a distance of 3.4 Å.

As for the binding mode of the lactone in the active site, hydrogen bonds were formed from O1 through O4 with K106 (O1 at 2.9 Å; distance to a counter-part oxygen), Y135 (O2 at 2.8 Å), Y163 (O3 at 3.1 Å), E165 (O3 at 2.5 and O4 at 2.7 Å), H195 (O1 at 2.9 Å), and H318 (O3 at 2.6 Å) (Fig 3A). The substrate was also sandwiched between F17 and L192 through hydrophobic interactions. Among these residues, H318 is located in the loop region of the adjacent subunit, which is a characteristic of the Pl-*scyllo*-IDH structure compared to other IDHs.

**Fig 4:**
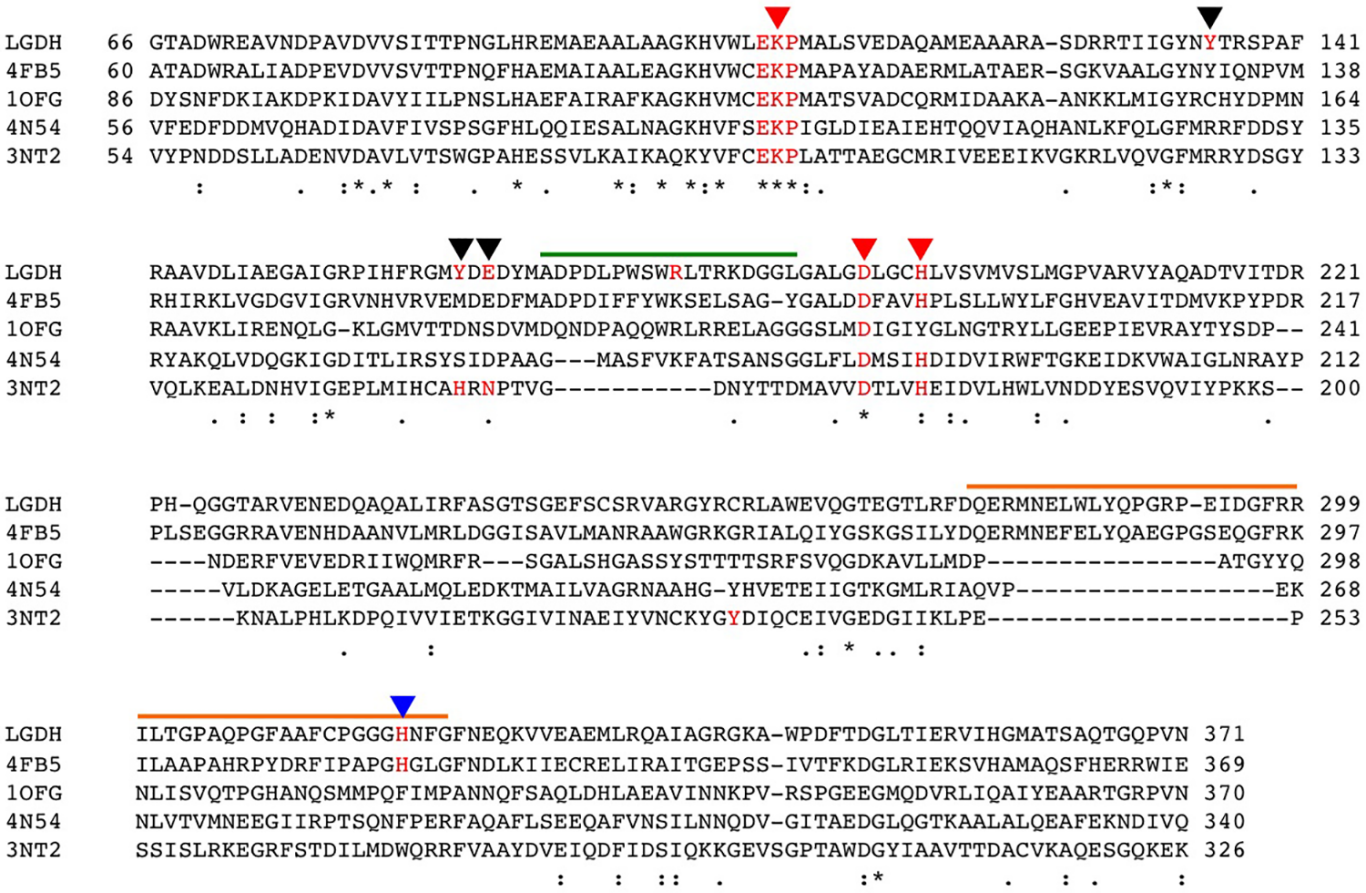
Sequence alignment of structurally identified GFO/IDH/MocA family proteins. The amino acid sequence alignment was performed using ClustalW. Proteins are represented by PDB codes as follows: 4FB5; a probable oxidoreductase from *Rhizobium etli*, 1OFG; a glucose-fructose oxidoreductase from *Zymomonas moblis*, 3NT2; a myo-IDH from *B*. *subtilis* and 4N54; a *scyllo*-inositol DH from *Lactobacillus casei*. The three red arrow heads show the proposed catalytic residues. The black arrow heads show residues involved in substrate binding in Pl-*scyllo*-IDH. The blue arrow head indicates H318 in Pl-*scyllo*-IDH, which is involved in the substrate binding in the adjacent subunit. The green line indicates the flexible loop region covering the active site in Pl-*scyllo*-IDH. Arg178 located in the loop region is colored in red. The orange line shows the loop region involved in the subunit interaction in Pl-*scyllo*-IDH.

As Pl-*scyllo*-IDH is a member of GFO/IDH/MocA family, it contains a conserved EKP motif (Fig 4). The residue K106 is in the middle of this motif, and forms a hydrogen bond to a carbonyl oxygen in the lactone ring. Among the seven residues described above, K106 and D191 are conserved in the GFO/IDH/MocA family, and H195 is conserved in the IDH family (Fig 4).

### Inositol derivative complex

**Fig 5:**
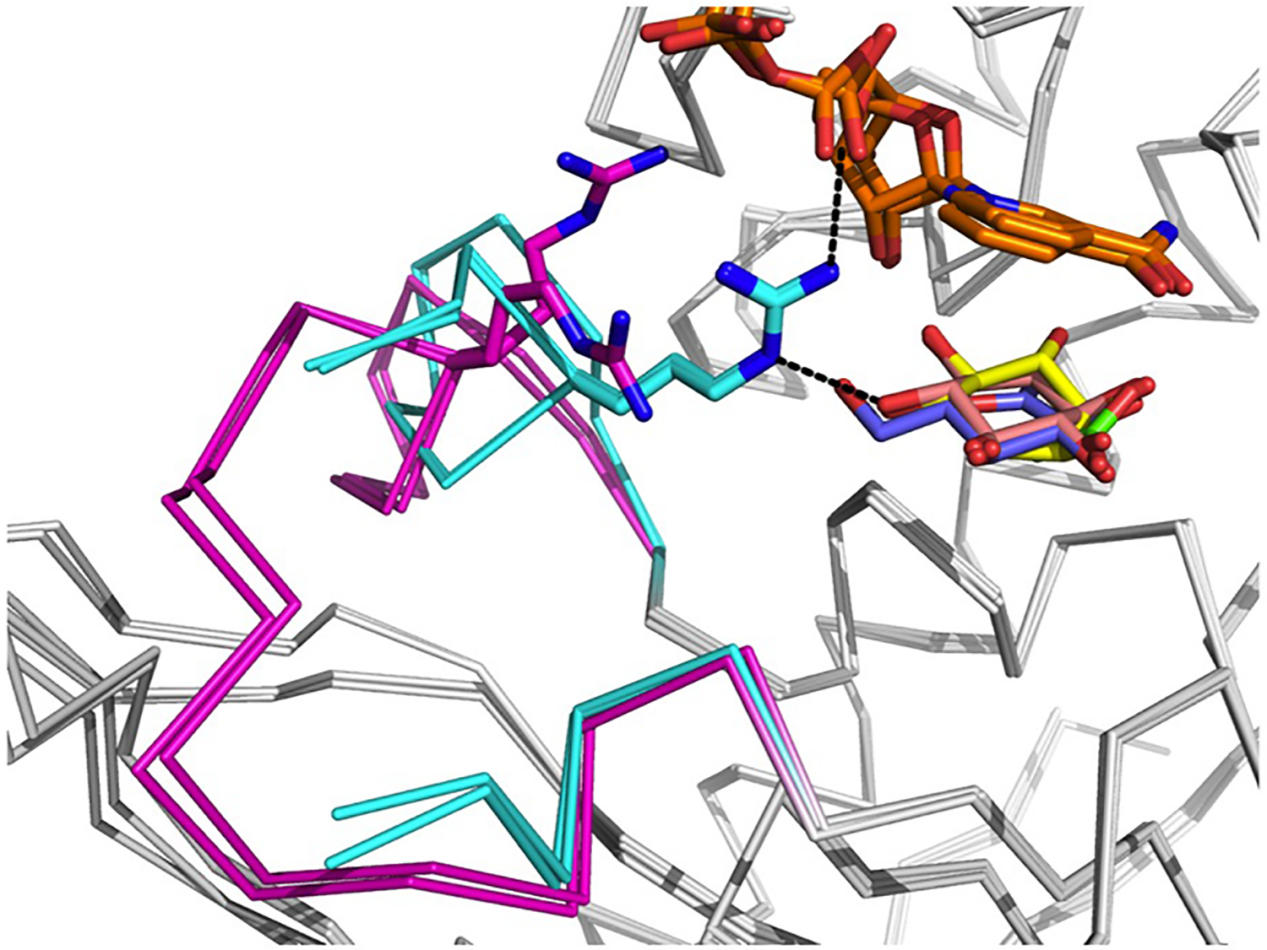
Superposition of flexible loop regions in each complex. The Pl-*scyllo*-IDH structures complexed with L-glucono-1,5-lactone, *myo*-inositol, *scyllo*-inosose, and acetate are shown superimposed, and the flexible loop regions are shown. Arg178 in the loop is also shown as a stick model. The regions colored in cyan and magenta indicate *myo*-inositol and *scyllo*-inosose, and L-glucono-1,5-lactone and acetate, respectively.

Since Pl-*scyllo*-IDH also oxidizes *scyllo*-inositol and *myo*-inositol, the complex structures of Pl-*scyllo*-IDH with *scyllo*-inosose and *myo*-inositol were also determined. Both inositols bound to the catalytic site in essentially the same manner as each other, where residues K106, Y135, Y163, E165, R178, D191, and H318 formed hydrogen bonds with the hydroxyl oxygen of the sugar. With the exception of R178 and D191, these residues were also involved in the binding of L-glucono-1,5-lactone. All six inositol hydroxyl oxygens were involved in hydrogen bonds, including bipartite and tripartite bonds making for a total of eleven bonds (Fig 3B and 3C). The mode of substrate recognition by R178 was remarkably different between the lactone and the inositols. The loop region, A169 to G185, which is not conserved in the other known *myo*-IDH structures (Fig 4), showed structural flexibility, and accordingly, R178 in the loop altered its position depending on the substrate. In case of L-glucono-1,5-lactone, R178 was located outside of the active site to avoid the protruding C6-hydroxyl group of the lactone ring. On the other hand, in the case of the inositols, R178 was positioned inside the active site in order to be able to interact with the substrates (Fig 5). Therefore, the flexibility of the position of R178 appears to contribute to the broad substrate specificity of Pl-*scyllo*-IDH.

In the case of *myo*-inositol binding, based on the shape of the electron density map, the 4’-OH formed a hydrogen bond with K106 (Fig 3B), which suggests that the reaction product is L-*epi*-2-inosose (Fig 1). *myo*-IDH from *B*. *subtilis* is known to oxidize 2ʹ-OH of *myo*-inositol resulting in *scyllo*-inosose production (Fig 1) [21]. Therefore, Pl-*scyllo*-IDH may have a different regioselectivity with *myo*-IDH toward *myo*-inositol. Then we performed a reverse reaction assay of Pl-*scyllo*-IDH using L-*epi*-2-inosose as a substrate. As shown in Table 3, Pl-*scyllo*-IDH exhibited NADH-dependent reductase activity for L-*epi*-2-inosose. These values are comparable to other substrates studied previously with Pl-*scyllo*-IDH (Table 3). Therefore, L-*epi*-2-inosose can be a substrate for Pl-*scyllo*-IDH, and the enzyme is highly possible to be the first enzyme catalyzing reversible oxidation of *myo*-inositol to L-*epi*-2-inosose.

**Table 3:** Kinetic parameters of Pl-*scyllo*-IDH with L-2-*epi*-inosose and substrates.

Substrate	*K*_m_ (mM)	*k*_cat_ (min^-1^)	*k*_cat_/*K*_m_ (min^-1^mM^-1^)
L-*epi*-2-inosose	40.3 ± 2.2	4500 ± 280	113
*myo*-inositol*	53.3 ± 8.6	572 ± 30	10.7
*scyllo*-inositol*	3.70 ± 0.4	705 ± 12	190
L-glucose*	59.7 ± 5.7	1040 ± 28	17.4

*Values are obtained in the previous study [4]

In the case of *scyllo*-inosose bound to Pl-*scyllo*-IDH, the position of the carbonyl oxygen was determined based on the electron density of the Fo-Fc omit map (Fig 3C), and the carbonyl oxygen located between K106 and H195 formed hydrogen bonds, with distances of 3.2 Å and 2.9 Å, to K106 and H195, respectively (Fig 3C). The complex structure with *scyllo*-inosose has been reported for *myo*-IDH from *B*. *subtilis*. In this complex, D172 (corresponding to D192 in Pl-*scyllo*-IDH) is not involved in the binding of *scyllo*-inosose, since its side chain is parallel to the plane of the sugar ring, as has been observed in the Pl-*scyllo*-IDH structure complexed with the lactone. On the other hand, in the Pl-*scyllo*-IDH structure complexed with *scyllo*-inosose, D192 formed hydrogen bonds to the hydroxyl groups of *scyllo*-inosose, as was observed in the case of the *myo*-inositol complex of Pl-*scyllo*-IDH (Fig 3C).

Pl-*scyllo*-IDH has a lower *K*_m_ value for *scyllo*-inositol than that for *myo*-inositol [4], although the *k*_cat_ values are almost same. All six of the hydroxyl groups in *scyllo*-inositol adopted an equatorial conformation; on the other hand, one hydroxyl group in *myo*-inositol adopted an axial conformation (Fig 1). Therefore, *scyllo*-inositol can be accepted in the catalytic site of Pl-*scyllo*-IDH in any direction, and this may contribute to its lower *K*_m_.

### Enzymatic activity

A catalytic triad consisting of K-D-H has been identified in the inositol dehydrogenase family [8, 9, 22, 23]. Based on the crystal structure of Pl-*scyllo*-IDH complexed with inositols, side chains of D191 and H195 were found to be oriented to K106; on the other hand, in the structure complexed with L-glucono-1,5-lactone, the side chain of D191 was parallel to the sugar plane and extended outward from the active site. Therefore, to confirm whether these conserved residues in Pl-*scyllo*-IDH are important for the catalytic activity, we prepared single amino acid mutants of 3 residues substituted to alanines, which resulted in a loss of enzyme activity (data not shown) as expected. Therefore, in the complex with lactone, a water molecule between K106 and D191 may be involved in a proton relay system that is part of the catalytic mechanism, where distances were 3.1 Å (K to the water molecule) and 3.5 Å (D to the water molecule) in chain A, 2.9 Å and 2.8 Å in chain B, and 3.3 Å and 3.0 Å in chain C, respectively.

Based on the substrate recognition modes revealed by the crystal structures, in addition to the three proposed catalytic residues, R178 and H318 were unique residues compared to residues in other known IDHs. Therefore, we also generated the single-amino acid substituted mutants R178A and H318A. The results revealed that both mutations showed different effects (Table 4). R178A increased the *K*m value by 10-fold against wild type and decreased the *k*cat value for *scyllo*-inositol as a substrate. Since R178 bound to both *scyllo*-inositol and NADH (Fig 5), the alanine mutation lost these connections resulting in lower activity. Therefore, R178 is necessary for the full activity with *scyllo*-inositol as a substrate. On the other hand, when L-glucose was used as a substrate, *K*m value increased, and *k*cat value also increased by approximately 5-fold against wild type. Although R178 formed no interactions with either L-glucose or NADH on the basis of the crystal structure (Fig 5), the result may suggest some interactions exist between R178 and L-glucose and/or NAD^+^ in solution. If R178 could interfere with the C6 group of L-glucose, the product release from the active site may faster in R178A mutant. In case of H318A mutant, the *k*cat value for *scyllo*-inositol decreased resulting in a very weak activity. Furthermore, when L-glucose was used as a substrate, the activity was so low that we were unable to obtain kinetics parameters. H318 is located in the loop from the next subunit and is involved in the substrate binding for both *scyllo*-inositol and L-glucose. Based on the crystal structures complexed with substrates, H318 appeared not to be critical in the substrate binding, as other two residues, Y163 and E165, formed hydrogen bonds with the same hydroxyl oxygen of substrates as H318 (Fig 3). Therefore, further analyses are needed to understand these results.

**Table 4:** Kinetic parameters of Pl-*scyllo*-IDH mutants with *sycllo*-inositol and L-glucose.

	*scyllo*-inositol			L-glucose		
	*K*m (mM)	*k*cat (min^-1^)	*k*cat/*K*m (min^-1^mM^-1^)	*K*m (mM)	*k*cat (min^-1^)	*k*cat/*K*m (min^-1^mM^-1^)
WT*	3.70 ± 0.4	705 ± 12	190	59.7 ± 5.7	1040 ± 28	17.4
R178A	40.6 ± 7.2	486 ± 39	12.0	98.1 ± 3.9	4856 ± 158	49.5
H318A	154 ± 43	85.6 ± 21	0.56	n.a.	n.a.	n.a.

*The values are obtained from the previous study [4]. n.a. denotes that data not available.

Interestingly, in the apo structure, an acetate molecule originating from the crystallization solution was also found to be bound in the active site. This molecule formed hydrogen bonds with Y135, Y165, E195, and H318, which were the same residues used for substrate binding, other than the three catalytic residues, which suggests that these 4 residues may only be required for substrate binding, although the role of H318 remains unclear as described above.

**Fig 6:**
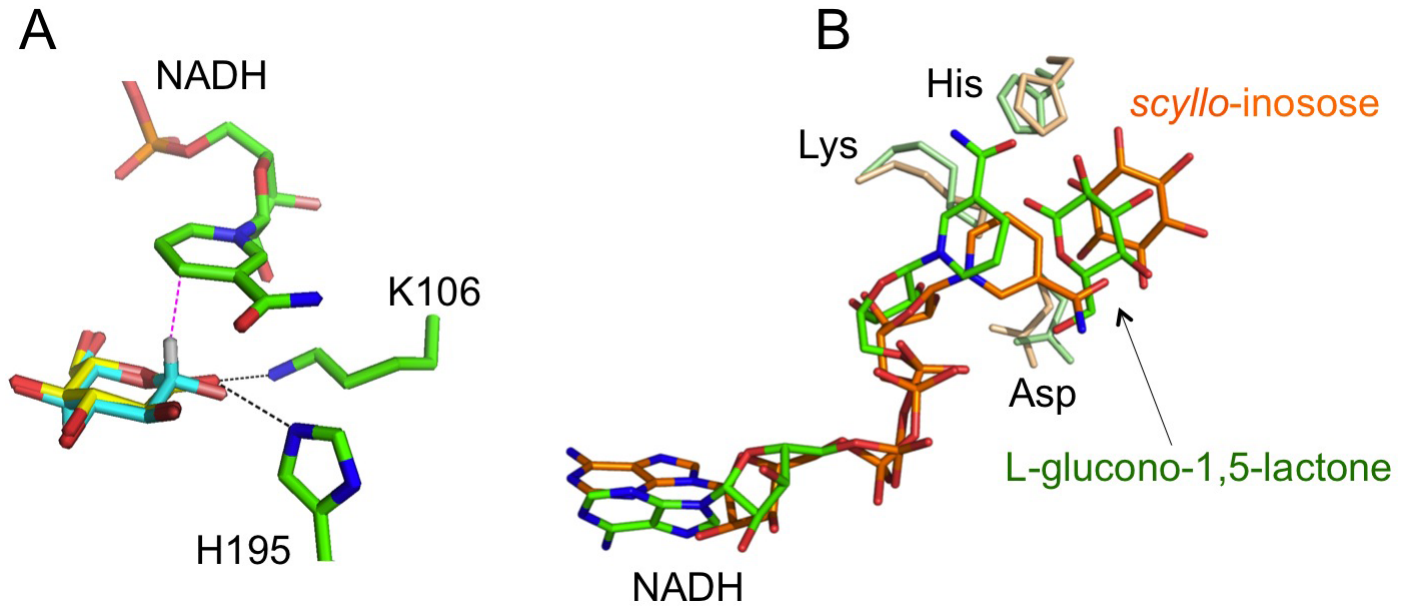
Binding mode of NADH. (A) L-glucono-1,5-lactone (yellow) and NADH (green) bound in Pl-*scyllo*-IDH are shown as stick models. β-L-glucose, colored in cyan, is superposed on the lactone molecule, and the H1 hydrogen in the axial conformation is colored white. The distance between NC4 and C1 is 3.4 Å, and the distance between H1 and NC4 is 2.0 Å shown as a red dotted line. From this structure, Pl-*scyllo*-IDH is concluded to be an A-type enzyme. (B) Structures of each of chain B in Pl-*scyllo*-IDH complexed with L-glucono-1,5-lactone colored in green and pale green, and *myo*-IDH from *B*. *subtilis* complexed with *scyllo*-inosose (PDB code: 3NT5) colored in orange and pale orange were superposed using the program Chimera [25]. The superposed active sites are shown with substrates, NADH, and the catalytic triad, K-D-H in stick models. The difference is observed in the orientations of the nicotinamide rings between Pl-s*cyllo*-IDH and *myo*-IDH, which represent the A-type and B-type enzymes, respectively.

As for hydride transfer of the C1 hydrogen to NAD^+^, in the complex of Pl-*scyllo*-IDH with the lactone, the nicotinamide ring of NADH was found to be stacked on top of the lactone ring, where the C4 atom of the nicotinamide ring of NADH was aligned over the C1 atom of the lactone at a distance of 3.4 Å (Fig 6A). With this conformation, the C1 hydrogen atom at the axial position in β-L-glucose would be transferred to the *R* side of the C4 atom of NAD^+^, therefore, Pl-*scyllo*-IDH is considered an A-type enzyme. The corresponding distances in the *myo*-inositol and *scyllo*-inosose complexes were 2.8 Å and 3.1 Å, respectively, and the relative positions of NAD^+^ and the inositols were the same as those seen in the lactone complex. Alizade et al. previously demonstrated, using an isotope labeling experiment, that *myo*-IDH from *Klebsiella pneumoniae* is an A-type enzyme [24], whose structure is unknown. On the other hand, van Straaten et al. reported that *myo*-IDH from *B*. *subtilis* is a B-type enzyme similar to SDR proteins, which use pro-*S* hydrogen, based on the crystal structure in complex with *scyllo*-inosose [8] (Fig 6B). Therefore, this may also be one of the characteristics that distinguish Pl-*scyllo*-IDH from other known *myo*-IDHs.

### Phylogenetic study

**Fig 7:**
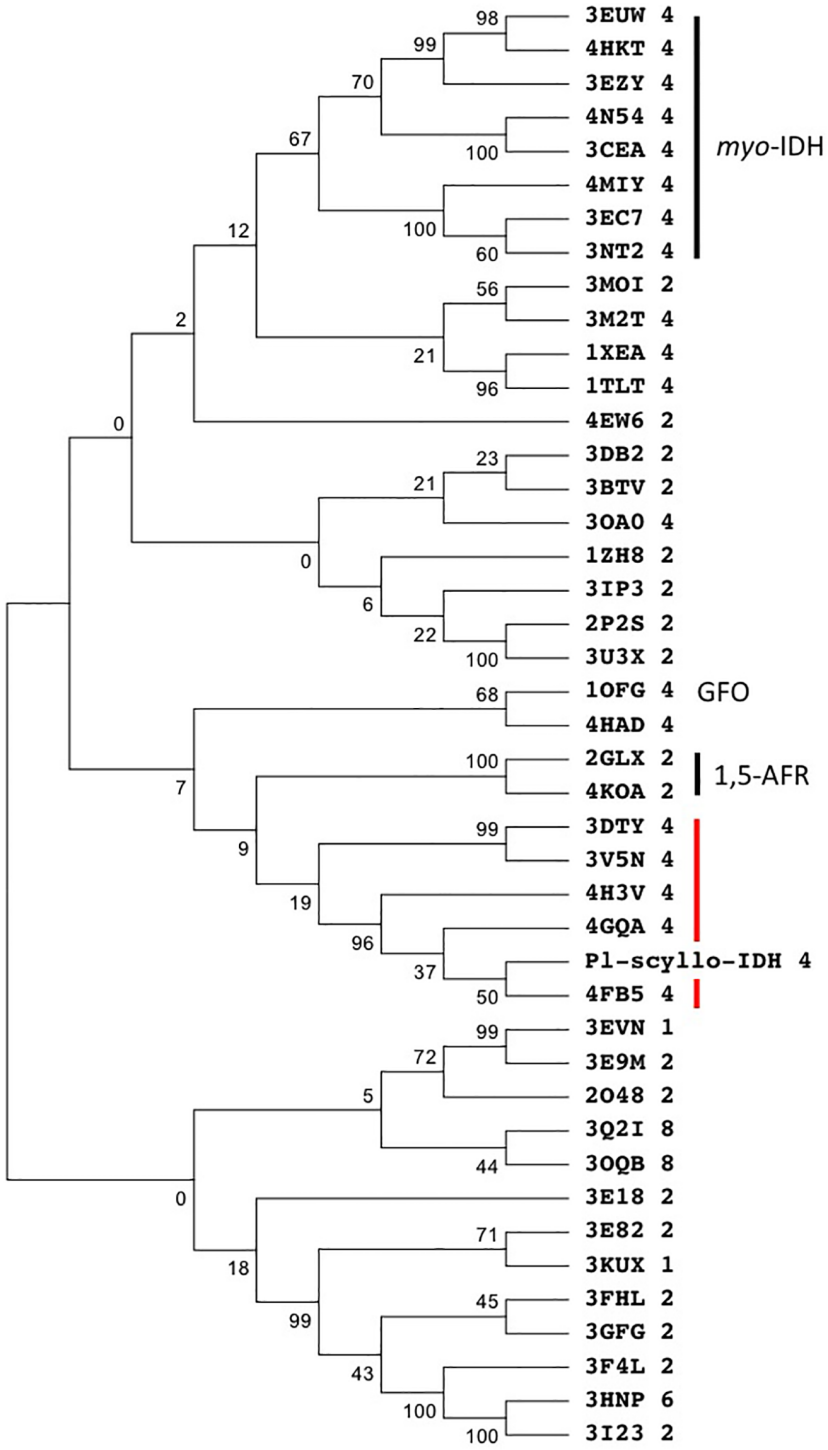
Phylogenetic tree of GOFR/IDH/MocA family members present in PDB. The amino acid sequences of GFO/IDH/MocA family enzymes in PDB were aligned and then subjected to a phylogenetic analysis using the program MEGA7. The number after PDB code indicates the oligomeric state of enzyme. A red line shows structures harboring the swap loop region as observed in Pl-*scyllo*-IDH.

When the amino acid sequence of Pl-*scyllo*-IDH was subjected to a BLAST search, we obtained a list of *myo*-IDHs, including one from *B*. *subtilis*. Therefore, we expected that the structure of Pl-*scyllo*-IDH would be similar to that of *myo*-IDH from *B*. *subitlis*, which is characterized in detail [23, 26]. However, the crystal structure of Pl-*scyllo*-IDH and that of *myo*-IDH from *B*. *subtilis* mainly differ in the mode of subunit interactions (Fig 2). Furthermore, the overall structure of Pl-*scyllo*-IDH was rather similar to GFOR from *Zymmomonas mobilis*. Therefore, structures deposited in PDB, which were categorized as containing the Pfam_GFO/IDH/MocA domain, were used to perform a phylogenetic analysis using the MEGA7 software. As shown in Fig 7, the Pl-*scyllo*-IDH and PDB entries 4FB5, 4GQA, 4H3V, 3DTY and 3V5N, which were the top hits from the Dali search, were found to be in the same clade. With the exception of 4GQA, all of these structures have the same swapped loop like Pl-*scyllo*-IDH. 4GQA also has a loop region similar to that seen in Pl-*scyllo*-IDH, but it does not loop out to interact with the active site in the adjacent subunit. On the other hand, *myo*-IDHs, including one (3NT2) from *B*. *subtilis*, were clustered into a clade different from that of Pl-*scyllo*-IDH. Most of the structures listed in Fig 7 have been deposited in PDB without an accompanying publication, therefore, it is difficult to judge whether the phylogenetic tree corresponds to substrate specificity. However, based on our data examining Pl-*scyllo*-IDH substrate specificity, showing that it is a *scyllo*-inositol dehydrogenase with a broad substrate specificity, and the phylogenetic analysis, Pl-*scyllo*-IDH could represent a different subfamily distinct from that of known *myo*-IDHs.
